# Inflammatory and oxidative stress markers in intracerebral hemorrhage: Relevance as prognostic markers for quantification of the edema volume

**DOI:** 10.1111/bpa.13106

**Published:** 2022-06-28

**Authors:** Vladimir Rendevski, Boris Aleksovski, Ana Mihajlovska Rendevska, Nikola Hadzi‐Petrushev, Nensi Manusheva, Blagoj Shuntov, Icko Gjorgoski

**Affiliations:** ^1^ Medical Faculty Ss. Cyril and Methodius University in Skopje Skopje North Macedonia; ^2^ Faculty of Natural Sciences and Mathematics‐Skopje Institute of Biology, Ss. Cyril and Methodius University in Skopje Skopje North Macedonia

**Keywords:** brain edema, hemorrhagic stroke, neuroinflammation, oxidative stress markers

## Abstract

We aimed to analyze the inflammatory and oxidative stress (OS) markers after intracerebral hemorrhage (ICH) and their temporal changes, interaction effects, and prognostic values as biomarkers for the prediction of the edema volume. Our prospective, longitudinal study included a cohort group of 73 conservatively treated patients with ICH, without hematoma expansion or intraventricular bleeding, which were initialized with the same treatment and provided with the same in‐hospital care during the disease course. Study procedures included multilevel comprehensive analyses of clinical and neuroimaging data, aligned with the exploration of 19 inflammatory and five OS markers. White blood cells (WBC), C‐reactive protein (CRP), erythrocyte sedimentation rate (ESR), neutrophilia, and lymphopenia peaked 3 days post‐ICH, and they showed much stronger correlations with clinical and neuroimaging variables, when compared to the admission values. An intricate interplay among inflammatory (WBC, CRP, neutrophils, neutrophil‐to‐lymphocyte ratio [NLR], interleukin (IL)‐6, and IL‐10) and OS mechanisms (catalase activity and advanced oxidation protein products [AOPP]) was detected operating 3‐days post‐ICH, being assessed as relevant for prediction of the edema. The overall results suggested complex pathology of formation of post‐ICH edema, via: (A) Not additive, but statistically significant synergistic interactions between CRP‐ESR, neutrophils‐CRP, and neutrophils‐IL‐6 as drivers for the edema formation; (B) Significant antagonistic effect of high protein oxidation on the CRP‐edema dependence, suggesting a mechanism of potential OS‐CRP negative feedback loop and redox inactivation of CRP. The final multiple regression model separated the third‐day variables NLR, CRP × AOPP, and WBC, as significant prognostic biomarkers for the prediction of the edema volume, with NLR being associated with the highest effect size. Our developed mathematical equation with 3D modeling for prediction and quantification of the edema volume might be beneficial for taking timely adequate strategies for prevention of delayed neurological deteriorations.

## Graphical abstract

The complex formation of post‐ICH brain edema.

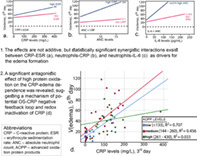

## 1 INTRODUCTION

Intracerebral hemorrhage (ICH), a detrimental disease, is associated with 30‐day case fatality of 40.4% and, among survivors—with the worst long‐term functional outcomes [1]. Over the past decades, emerging evidence suggests that if hematoma expansion and intraventricular bleeding are excluded, the main driver for delayed neurological deterioration post‐ICH is the formation and “evolution” of the brain perifocal edema [2], which contributes to at least 75%‐increase in the perihematomal volume [3]. The formation of edema can severely worsen patient's condition and recovery, as a result of its mass effect, leading to increased intracranial pressure, herniations, and neurological deficit [2, 3]. Today, the edema volume is considered as notable neuroimaging marker for secondary brain injury and a well‐known predictor for poor outcome after ICH [4, 5, 6].

Hence, aiming at prevention of formation of large edema volumes in patients after ICH, several studies up to now focused on its prediction. Most of these studies point out to the primary role of inflammatory mechanisms in the formation of large edema volumes; for instance, our previous work stressed the importance of TNF‐α, a pro‐inflammatory cytokine, in the formation of the brain edema [7], as well as its role in prediction of the 3‐month functional outcome [8]. Nevertheless, the data concerning the role of other cytokines in brain edema formation and functional outcome after ICH is scarce.

A vast number of scientific data shows the mutual connection between neuroinflammation and oxidative stress (OS) in brain injury [9], but the inter‐relation and inter‐dependence between inflammatory and OS markers specifically in the ICH setting were not studied in details. Moreover, their relation to the neuroimaging computer tomography (CT) findings and the severity of symptoms was evaluated only in several studies.

Consequently, this study aimed at exploration of the effects, the interplay, and inter‐dependence among inflammatory and OS markers in ICH patients, with a special focus to the main drivers in formation of the brain edema post‐ICH.

## 2 METHODS

### 2.1 Study design and cohort patients

We performed prospective, longitudinal cohort study in the period from March 2019 to May 2020, focused on conservatively treated non‐comatose patients with acute, primary, supratentorial ICH, without hematoma expansion or intraventricular bleeding during the disease course, which were followed from admission to their hospital discharge.

During the defined period, all patients suspected for ICH, admitted to the emergency department triage, were included in the initial screening. Brain CT scan was performed, and all patients positive for ICH were considered as potentially eligible. To evaluate their eligibility, the following inclusion criteria were implemented: patients diagnosed for acute, primary, supratentorial ICH, without severe alternation of the consciousness (unarousable unresponsiveness—deep coma), which were hospitalized during the first 4 h after symptom onset. Exclusion criteria were: of previously diagnosed cerebrovascular insult, and presence of comorbidities (neurological and neurodegenerative disorders, pulmonary, renal or hepatic disorders, chronic inflammatory or autoimmune disorders, vascular malformations or aneurisms, coagulopathies, or intratumoral hemorrhage). All patients were tested for COVID‐19; positive patients for COVID‐19 were excluded from the study.

All eligible patients entered the study and depending on their clinical state they were hospitalized in the intensive care units within the University Clinic of Neurology or, Neurosurgery. At admission, all in‐patients were initialized with the same conservative treatment (osmotic, anti‐hypertensive, neuroprotective, and symptomatic therapy) according to the provided guidelines [10], and their clinical trajectory during the hospitalization was studied in details. During the follow‐up, patients with serious deterioration as a result of hematoma expansion, intraventricular or subarachnoid bleeding, which required surgical interventions, were excluded from the study. Patient's early outcome was registered at hospital discharge (usually within 3 weeks after admission) as survivors or patients with in‐hospital mortality.

The reporting of this study conforms to STROBE cohort reporting guidelines [11].

### 2.2 Study procedures: Clinical, radiological, and biochemical analyses

At admission (during the first 4 h post ICH‐onset), patient's age, sex, blood pressure, heart rate and previous diagnosed diseases were recorded, their neurological state at admission was evaluated in details and, scored by the Canadian Stroke Scale (CSS). Additionally, the first CT scan was performed at admission, and the anatomic localization and hemispheric side of the ICH was recorded; the initial volume of the hematoma was also assessed by the formula V = A × B × C/2, as previously described [7]. The second CT scan was performed on the 5th day (app. 120 ± 2 h post‐ICH), and the volumes of the hematoma and the perifocal edema were calculated. The edema was defined as the most hypodense area immediately surrounding the ICH and more hypodense than the corresponding area in the contralateral hemisphere; the volume of the edema was determined by subtracting the volume of the ICH from that of the total lesion, as previously described [7].

Blood samples for analyses were taken serially (at admission, 1‐, 2‐, 3‐, and 5‐days post‐ICH), and the white blood cell count (WBC) along with the differential leukocyte count (absolute neutrophil count [ANC], absolute monocyte count [AMC], absolute lymphocyte count [ALC]) were determined by 5‐Part Differential Cell Counter. Additionally, serial measurements were performed for plasma C‐reactive protein (CRP) levels (by using CRP‐hs latex‐high sensitivity kit, BioSystems) and for erythrocyte sedimentation rate (ESR); blood glucose levels (Glucose Oxidase/Peroxidase kit, BioSystems) were assessed only at admission.

Since previous detailed research showed that the neuronal death, inflammation, OS, and the recruitment of infiltrating leukocytes peak between 1‐ and 3‐days post‐ICH [9], coinciding with observation of the highest levels of interleukins (IL) [12], the levels of IL‐6 and IL‐10, as well as the OS markers were measured in the blood plasma samples collected on the 3rd day (72 ± 2 h post‐ICH).

IL‐6 and IL‐10 were determined by ELISA (IL‐6 human high sensitivity kit from Enzo®, Cat. No. ENZ‐KIT178‐0001 and IL‐10 human ELISA Kit from Enzo®, Cat. No. ADI‐900‐036), according to manufacturer's instructions. OS markers were determined by kinetic spectrophotometric assays; the activity of catalase (CAT), superoxide dismutase (SOD) and glutathione peroxidase (GPx), as well as the malondialdehyde levels (MDA), were measured as previously described [13]; the levels of advanced oxidation protein products AOPP were determined according to Witko‐Sarsat [14].

### 2.3 Ethical statement

The study complied with the principles of the Declaration of Helsinki; approval of our local institutional review board was obtained (Human Research Ethics Committee within UKIM‐Medical Faculty in Skopje) and all patients gave informed consent.

### 2.4 Statistical analyses

The statistical analyses were performed in IBM SPSS Statistics® 21; additionally, JMP® 14 (Copyright © 2018 SAS Institute Inc.) was used for modeling purposes. All categorical variables are expressed as percentage of individuals. Most of the quantitative variables followed a normal distribution, and consequently, are reported as mean ± SD. The independent samples Student *t*‐test was used for comparison of the means among the groups, and the paired samples *t*‐test was used for comparison of the means of repeatedly measured samples.

The bivariate statistical analyses were performed by using parametric correlations with the Pearson *r*‐coefficient. Moderation analyses and interaction plots were built in order to assess effects of interaction. The prediction of the edema volume was performed by building multiple regression models, with a stepwise‐backward selection, using the standard least squares method. The 3D models were constructed by using the prediction formula of the multiple regression model and the contour profiler in JMP 14. In all cases, the level of statistical significance was defined as *p* < 0.05 (marked as *), that is, *р* < 0.001 for highly significant (**).

## 3 RESULTS

### 3.1 Basic patient characteristics

During the defined period, out of 345 potentially eligible patients, only 230 have fulfilled the initial screening to be examined for eligibility (excluded patients had negative CT finding for ICH [84 cases] or, did not give informed consent [31 cases]). After implementation of the inclusion and exclusion criteria, only 118 were confirmed eligible and 112 were excluded (38 parents had severe comorbidities, 43 were hospitalized later than 4 h from the ICH onset, 24 were hospitalized with deep coma at admission, 7 were positive for COVID‐19). During the follow up, 45 patients were excluded from the study (8 with hematoma expansion, 19 with intraventricular bleeding, 7 with subarachnoid bleeding, 11 with incomplete data), so finally 73 patients have completed the study and were included in the statistical analyses; their basic demographic, clinical and biochemical characteristics are summarized in Table 1 (note: admission variables express changes max to 4 h post‐ICH onset; variables on the 3rd day depict changes of 72 ± 2 h post‐ICH, and variables on the 5th day represent changes 120 ± 2 h post‐ICH).

**TABLE 1:** Patient characteristics

Variables	Frequency^a^/mean ± SD^b^
Basic and clinical characteristics	
Sex	
Male	63.0%
Female	37.0%
Age/years	71 ± 10
Neurological state at admission (scored by CSS)	5.4 ± 3.0
Presence of diagnosed *Diabetes mellitus*	26.0%
Presence of diagnosed hypertension	78.1%
Admission glucose levels/mmol L^−1^	8.9 ± 4.5
Admission blood pressure	
SBP/mmHg	180 ± 25
DBP/mmHg	99 ± 11
Admission heart rate/BPM	88 ± 16
In‐hospital mortality rate	28.8%
Neuroimaging CT markers	
Admission ICH volume/cm^3^	25.88 ± 19.94
ICH volume, 5th day/cm^3^	29.92 ± 27.11
Brain perifocal edema volume, 5th day/cm^3^	41.93 ± 33.26
Anatomic localization of ICH	
Lobar	42.5%
Deep	57.5%
Hemispheric side	
Left	58.9%
Right	41.1%
Inflammatory mediators (at admission and 3 days post‐ICH)	
Admission WBC/10^9^ L^−1^	9.0 ± 1.9
Admission leukocytosis (%)	17.8%
WBC, 3rd day/10^9^ L^−1^	12.5 ± 3.5
Leukocytosis, 3rd day	57.5%
Admission CRP/mg L^−1^	11.45 ± 13.37
CRP, 3rd day/mg L^−1^	88.17 ± 43.72
Admission ESR/mm h^−1^	25 ± 14
ESR, 3rd day/mm h^−1^	41 ± 22
ALC, 3rd day/10^9^ L^−1^	1.6 ± 0.7
Lymphocytosis, 3rd day	6.3%
Lymphopenia, 3rd day	43.7%
ANC, 3rd day/10^9^ L^−1^	9.7 ± 3.3
Neutrophilia, 3rd day	62.5%
AMC, 3rd day/10^9^ L^−1^	1.3 ± 1.0
Monocytosis, 3rd day	47.9%
LMR, 3rd day	1.9 ± 1.3
NLR, 3rd day	7.7 ± 4.1
IL‐6, 3rd day/pg mL^−1^	63.83 ± 60.60
IL‐10, 3rd day/pg mL^−1^	12.37 ± 10.37
Oxidative stress (OS) markers	
AOPP levels, 3rd day/μmol L^−1^	194.45 ± 83.69
CAT activity, 3rd day/U mg^−1^ Protein	2.33 ± 3.72
SOD activity, 3rd day/mU mg^−1^ Protein	43.76 ± 23.43
GPx activity, 3rd day/mU mg^−1^ Protein	0.466 ± 0.386
MDA levels, 3rd day/μmol L^−1^	7.46 ± 8.24

*Note*: Admission variables express changes max to 4 h post‐ICH onset; variables on the 3rd day depict changes of 72 ± 2 h post‐ICH, and variables on the 5th day represent changes 120 ± 2 h post‐ICH.

Abbreviations: ALC, absolute lymphocyte count; AMC, absolute monocyte count; ANC, absolute neutrophil count; AOPP, advanced oxidation protein products; CAT, catalase; CRP, C‐reactive protein; CSS, Canadian stroke scale; CT, computer tomography; DBP, diastolic blood pressure; ESR, erythrocyte sedimentation rate; GPx, glutathione peroxidase; ICH, intracerebral hemorrhage; IL, interleukin; LMR, lymphocyte to monocyte ratio; MDA, malondialdehyde; NLR, neutrophil‐to‐lymphocyte ratio; SBP, systolic blood pressure; SOD, superoxide dismutase; WBC, white blood cells.

^a^ The categorical variables are expressed as total frequency (% of individuals).

^b^ The continuous variables are expressed as mean ± SD.

The cohort group of 73 patients was characterized by predominantly male participants (63%), with a mean age of 71 years and mean admission CSS score of 5.4. The main etiology for ICH was diagnosed hypertension (78.1%). Most of the patients had deep ICH, predominantly in the left hemisphere. In‐hospital mortality rate was 28.8%.

### 3.2 Temporal changes in non‐specific inflammatory markers (WBC, CRP, ESR) during the disease course

**FIGURE 1:**
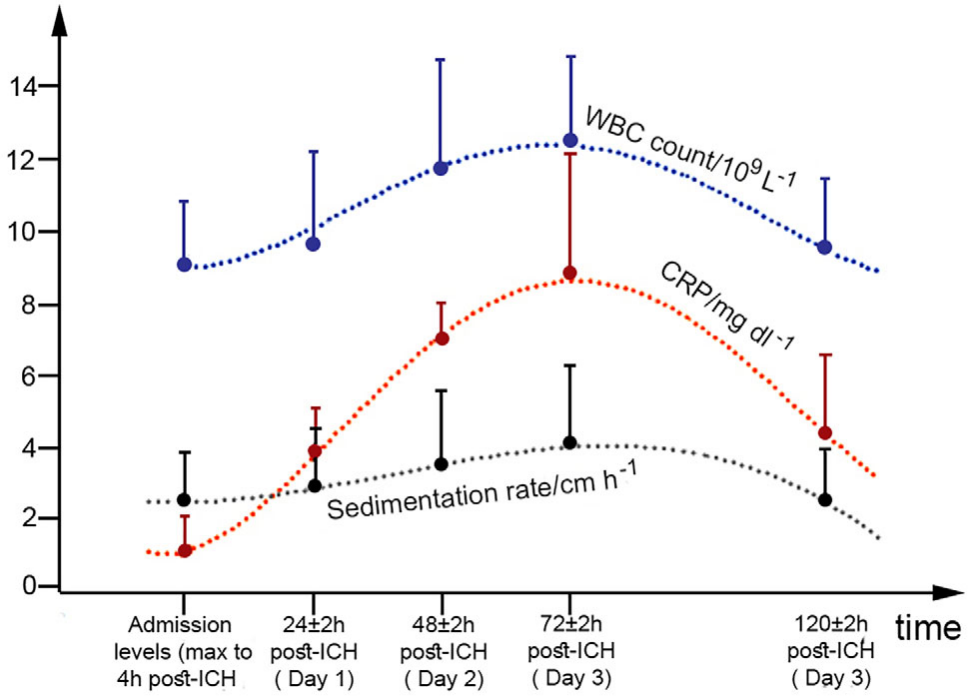
Trends of WBC, CRP, and ESR over time. Markers represent mean values, and bars SD. ESR is expressed as cm/h instead of mm/h and, CRP is given as mg/dl instead of mg/L in order to fit the same values of the scale. CRP, C‐reactive protein; ESR, erythrocyte sedimentation rate; WBC, white blood cells

The temporal changes in the non‐specific inflammatory markers (WBC, CRP, ESR), were followed during the disease course by repeated measurements (at admission, 1‐, 2, 3‐ and 5‐days post‐ICH), Figure 1. All of these markers followed similar trend, with increase of the values over the first 3 days and then, a steady decrease. The peak for each of these variables was detected on the 3rd day after admission. The paired‐samples *t*‐test also disclosed that the values of WBC (**p = 6.58 × 10^−15^), CRP (**p = 6.85 × 10^−13^), and ESR (**p = 0.00002) were significantly higher on the third day, when compared to the admission values.

### 3.3 Effects of sex, anatomic localization of ICH, hemispheric side, and presence of diagnosed hypertension and diabetes mellitus on the explored markers

The statistical analyses disclosed that neither sex nor hemispheric side affected the admission CSS score, the neuroimaging, inflammatory and the OS markers. The anatomic localization of ICH affected only the WBC, 3rd day (higher in deep ICH, *p = 0.005) and ANC, 3rd day (higher in deep ICH, *p = 0.026). The presence of diagnosed hypertension affected only the admission CSS score (lower in patients with hypertension, *p = 0.01) and the diagnosed *Diabetes mellitus* affected only the admission glucose levels (**p = 0.000003).

### 3.4 Correlation among variables

**FIGURE 2:**
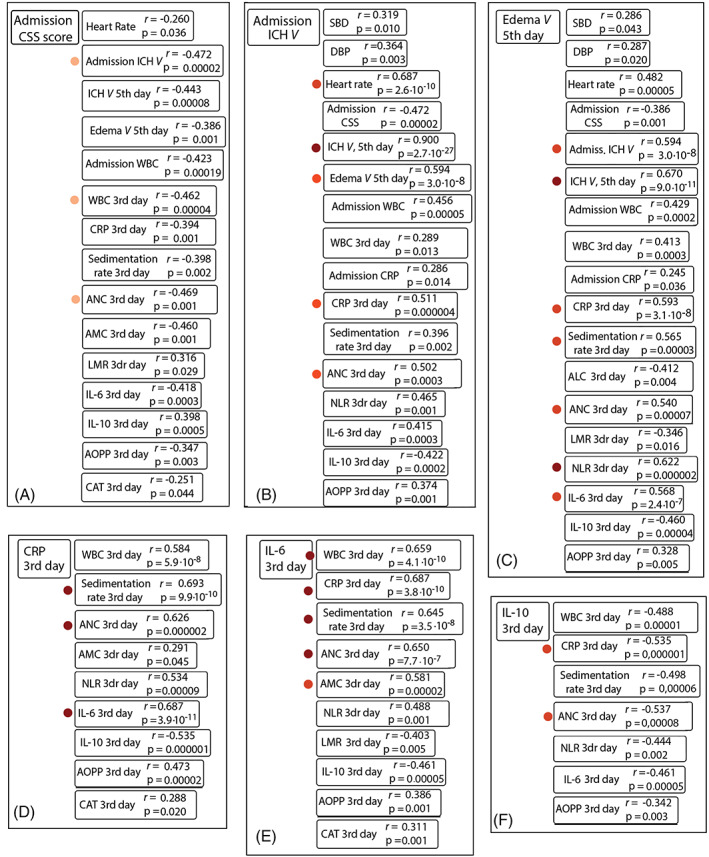
Correlations among variables. (A). Correlations with the neurological state (CSS scores) at admission. (B). Correlations with the admission ICH volume. (C). Correlations with the volume of the edema (5^th^ day). (D‐F). Mutual interplay among inflammatory and oxidative stress markers 3‐days post‐ICH. Abbreviations: ALC, absolute lymphocyte count; AMC, absolute monocyte count; ANC, absolute neutrophil count; AOPP, advanced oxidation protein products; CAT, catalase; CRP, C‐reactive protein; CSS, Canadian stroke scale; DBP, diastolic blood pressure; ICH, intracerebral hemorrhage; IL, interleukin; LMR, lymphocyte to monocyte ratio; NLR, neutrophil‐to‐lymphocyte ratio; SBP, systolic blood pressure; WBC, white blood cells

The variables Age, SOD, GPx, and Glucose levels did not correlate significantly with any of the other variables. Nevertheless, we found a number of significant correlations among the other variables; the most important correlations which depict the mutual interplay among inflammatory and OS markers are summarized in Figure 2.

The admission CSS score (Figure 2A) showed significant negative correlations with several variables, suggesting that in patients with worse neurological condition at admission (lower CSS), higher WBC, CRP, ESR, ANC, AMC and IL‐6 levels should be expected during the disease course.

The admission ICH volume (Figure 2B), correlated negatively with the CSS score, and showed very strong positive correlations with the edema and ICH volumes on the 5th day; it also correlated strongly with some of the inflammatory markers, suggesting that in patients with large hematoma, CRP, ANC, NLR, and IL‐6 should be elevated during the disease course. The negative correlation with IL‐10 demonstrates its protective role of subsiding inflammatory cascades in patients with large hematomas.

The volume of the edema (5th day) correlated with most of the variables (Figure 2C). Strong correlations were detected with the neuroimaging markers (admission ICH volume and ICH volume, 5th day); strong positive correlations were also detected for most of the inflammatory variables (admission WBC, and variables at 3rd day: WBC, CRP, ESR, ANC, NLR, and IL‐6), suggesting their potential role as drivers for formation of the edema. Moderate negative correlation was detected for IL‐10, thus suggesting a potential role of prevention in edema formation.

The panels (D), (E), and (F) of Figure 2 aim to summarize the mutual interplay among the inflammatory and OS markers 3 days post‐ICH. CRP (3rd day) correlated significantly with many other variables on the 3rd day, as WBC, ESR, ANC, and especially with IL‐6. A significant positive correlation was detected between CRP and AOPP, suggesting a possible effect of interaction between inflammatory and OS mechanisms.

The IL‐6 levels (3rd day) showed strong positive correlations with the WBC, CRP, ESR, ANC, and AMC 3 days after ICH. Moderate correlations were detected with NLR, LMR, AOPP, and CAT. Finally, IL‐10 showed negative correlations with most of the variables (WBC, CRP, ESR, ANC, NLR, IL‐6, AOPP), suggesting its protective role in secondary brain injury after ICH.

### 3.5 Multiple regression models for prediction and quantification of the edema volume

**FIGURE 3:**
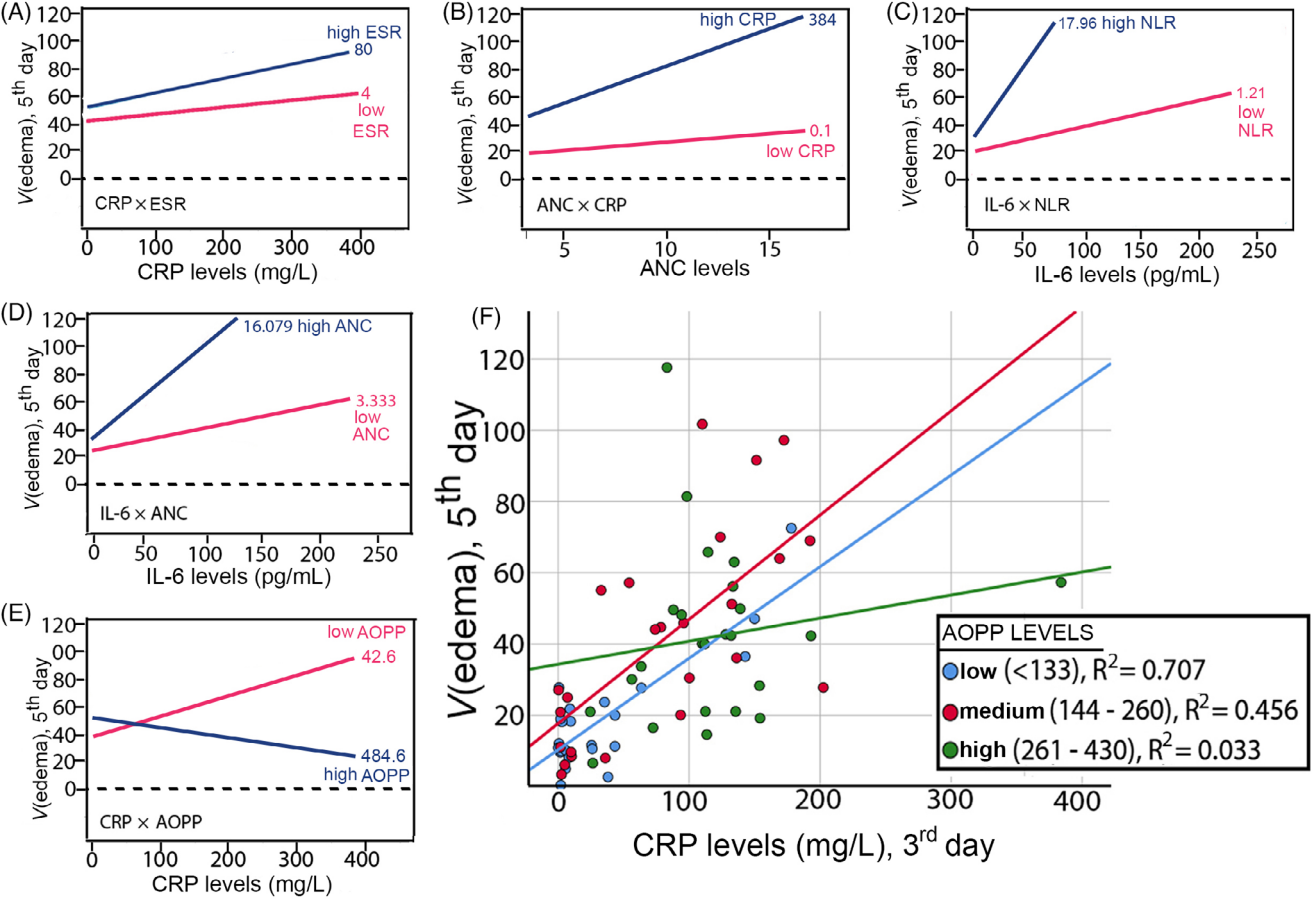
Effects of interaction between continuous variables on the edema volume. The overall results of the moderation analyses and the interaction plots summarise presence of not additive, but statistically significant synergistic interactions between CRP‐ESR (A), neutrophils‐CRP (B), NLR‐IL‐6 (C) and neutrophils‐IL‐6 (D) as drivers for the edema formation; moreover, significant antagonistic effect of high protein oxidation on the CRP‐edema dependence was detected (E‐F), suggesting a mechanism of potential redox‐CRP negative feedback loop and redox inactivation of CRP. Abbreviations: ANC, absolute neutrophil count; AOPP, advanced oxidation protein products; CRP, C‐reactive protein; ESR, erythrocyte sedimentation rate; IL, interleukin

Before building the multiple regression model for prediction of the edema volume, we tested the possibility for effects of interaction. A moderation analyses was performed, which showed five significant effects of interaction on edema volume: CRP × ESR (*p = 0.0276), ANC × CRP (*p = 0.0241), IL‐6 × NLR (*p = 0.0009), IL‐6 × ANC (*p = 0.0066), and CRP × AOPP (*p = 0.0008), Figure 3A–E. (note: all variables refer to 3 days post‐ICH.)

The overall analyses disclosed that the interactions between CRP‐ESR, ANC‐CRP, IL‐6‐NLR, and IL‐6‐ANC toward the edema volume were not additive, but more complex and synergistic. The dependence of the edema volume from ANC was increasing under conditions of higher CRP, while the volume of the edema was strongly affected by IL‐6 levels only when the NLR (c) and ANC (d) were high. The highest effect of interaction was detected between CRP and AOPP, which showed clear antagonism (e). AOPP levels acted as a significant moderator that can “buffer” the strength of the edema‐CRP dependence, since the correlation of CRP with the edema was clearly changing, as the levels of AOPP were changing (Figure 3F); only under the conditions of low AOPP, the volume of the edema was affected by the CRP levels, but this dependence was not present in conditions of high protein oxidation.

After identifying significant interaction effects, we proceeded to generate the multiple regression model. Since the variables age, AMC, CAT, SOD, GPx, MDA, and glucose levels did not correlate significantly with the edema volume, these variables were not initially included. Categorical variables as sex, anatomic localization of ICH, hemispheric side, diagnosed *Diabetes mellitus* and hypertension were also excluded, since previous analyses showed absence of any significant effects on the edema volume. The multiple regression modeling was performed with a stepwise‐backward selection by using the standard least squares method. Initially, all 18 variables which showed significant correlations (Figure 2C) and the five moderators (Figure 3) were included in the analyses; several cycles were performed, with step‐by step backward‐elimination of the variable with the highest *p* value; the final, optimal multiple regression model characterized by the highest *R* ^2^_Adj_ is shown in Table 2, consisting of seven independent variables, four of them estimated as significant predictors.

**TABLE 2:** Multiple regression model for prediction of the edema volume

Model effects	*B* ^a^	*p*	*β* ^b^
NLR, 3rd day	2.5058	0.0041*	0.3475
Admission heart rate	0.5214	0.0154*	0.2798
CRP × AOPP (3rd day)	−0.1010	0.0397*	−0.2202
WBC, 3rd day	2.5175	0.0467*	0.2923
IL‐6, 3rd day	0.1841	0.0561	0.3233
Admission CSS	−1.9002	0.1567	−0.1660
CRP, 3rd day	0.0766	0.2104	0.1940
Intercept	1.8774	0.9306	0

*Note*: The bold values represent significant effects (*p* < 0.05), marked with *.

Abbreviations: AOPP, advanced oxidation protein products; CRP, C‐reactive protein; CSS, Canadian stroke scale; IL‐6, interleukin‐6; NLR, neutrophil‐to‐lymphocyte ratio; WBC, white blood cells.

^a^ *B*—unstandardized coefficient.

^b^ *β*—standardized coefficient.

The final model included the moderator CRP × AOPP as a significant predictor, having higher effect in the overall model, when compared to the effects of the two variables alone. This effect of interaction was negative, as demonstrated by the previous moderation analyses (Figure 3F).

In the model, the admission heart rate, and the 3rd day variables NLR, the moderator CRP × AOPP and WBC, were estimated as significant predictive risk factors for formation of large edema volumes; among them, NLR (3rd day) showed the largest effect size, with 1 unit increase of NLR associated by 2.5 increase of the edema volume.

The following formula for prediction of the edema volume can be defined, based on the model:

*V*(edema, 5th day) = 2.5058∙(NLR 3rd day) + 0.5214∙(admission heart rate) – 0.1010∙(CRP × AOPP 3rd day) + 2.5175∙ (WBC 3rd day) + 0.1841∙(IL‐6 3rd day) – 1.9002∙ (admission CSS) + 0.0766 (CRP 3rd day) + 1.8774.

The overall predictive capacity of the model was estimated as moderate‐to‐high (62.12% of the variation in the volumes of the edema was accounted by the defined seven model effects; *R* ^2^ = 0.6814, *R* ^2^_Adj_ = 0.6212), and several statistical tests proved that the constructed model has good overall characteristics and fit (Figure S1).

**FIGURE 4:**
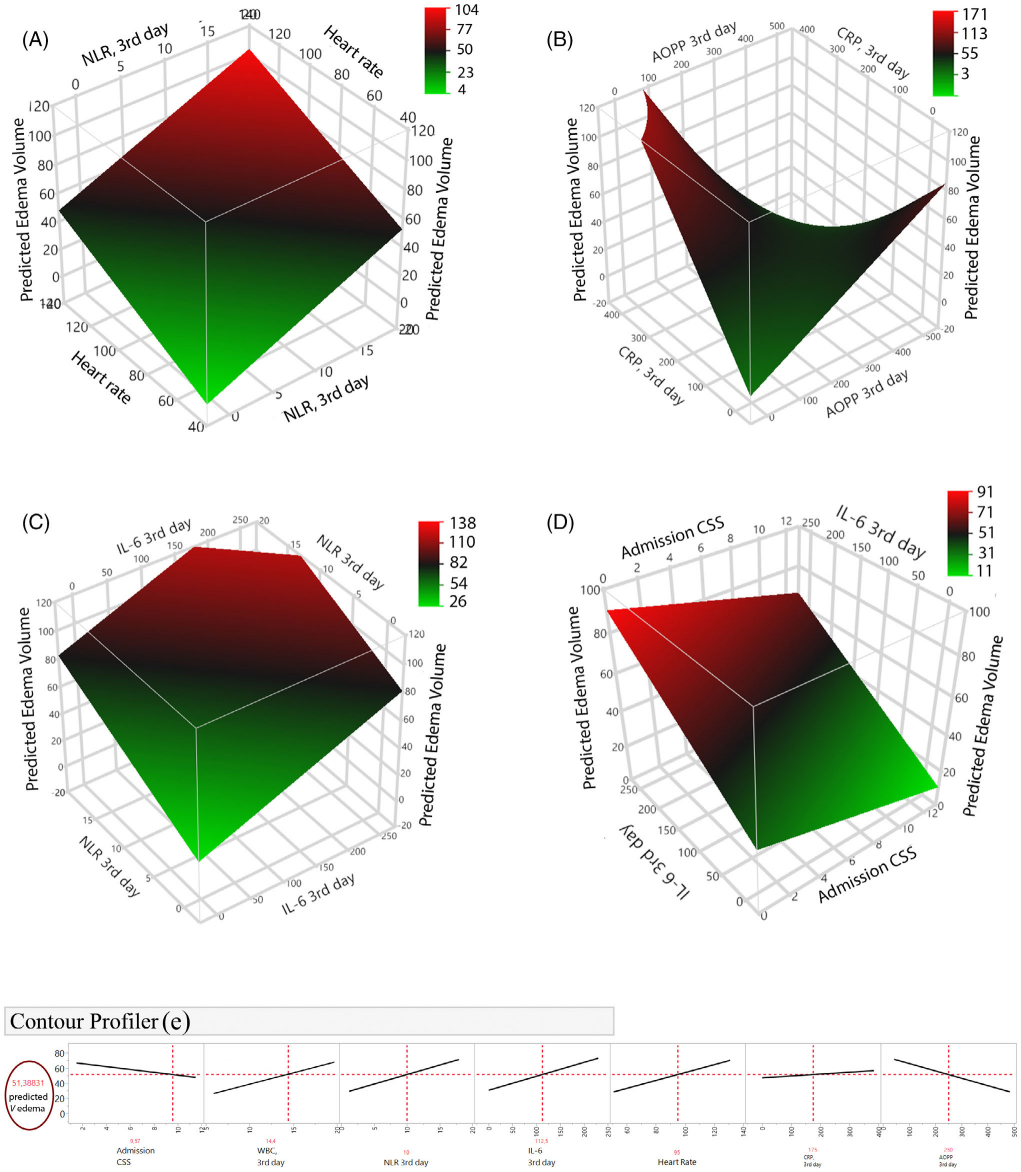
3D plots for prediction of the edema volume, based on the multiple regression model. The plots summarize four cases of the prediction capacity of the model to accurately predict the edema volume. (A) 3D plot of the effects of increased heart rate and increased NLR (3rd day) in prediction of the edema volume. (B). 3D plot modelling the buffering effect of AOPP high levels on the slope between CRP and edema volume. (C) 3D plot modelling the synergism between NLR and IL‐6 on the 3rd day for prediction of the edema volume. (D). Antagonistic effect between CSS and IL‐6 in prediction of the volume. Abbreviations: AOPP, advanced oxidation protein products; CRP, C‐reactive protein; CSS, Canadian stroke scale; IL, interleukin; NLR, neutrophil‐to‐lymphocyte ratio

Based on prediction formula, we also developed several interactive 3D plots (Figure 4A–D), that summarize four cases of the prediction capacity of the model to accurately predict the edema volume. These plots are only examples which depict two model effects per plot, while the other variables are kept constant by their mean values; however, the surface plots can also be largely influenced if these other variables are specified in the profiler. Nevertheless, the relation and slope of the model effects to the edema volume can be clearly observed; for instance, the 3D plot on Figure 4B distinctly shows the buffering effect of AOPP high levels on the slope between CRP and edema volume. The negative slope of AOPP in the contour profiler (Figure 4E) confirms this finding, also detected by previous analyses.

## 4 DISCUSSION

### 4.1 Non‐specific inflammatory markers during the disease course: Temporal changes, inter‐dependence, and correlations with clinical and neuroimaging markers

The WBC count, leukocytosis, neutrophilia, lymphopenia, CRP, and ESR were all repeatedly confirmed in the setting of the post‐ICH pathophysiology as part of the key inflammatory mechanisms that drive the notorious secondary brain injury; these insights were provided from both basic research in animal ICH models and, from a vast number of clinical studies.

Since clinical studies separated the acute leukocytosis as the first, most typical response to the hemorrhage [9, 15], peripheral WBC count was suggested as a marker which can reflect the activation of the inflammatory cascades after ICH. Similarly, CRP [16], and ESR were suggested as systemic biomarkers for neuroinflammation after ICH.

Nevertheless, many studies in the last decade focused on the temporal changes and the kinetics of these inflammatory markers after ICH [16, 17], stressing the question when should we take the sample [18]. While the initial injury and hemorrhage happens within minutes, the subsequent inflammatory response which contributes to damage of the surrounding tissue lasts for days [9]. Therefore, recent studies indicate the benefit of repeated (serial) measurements of the inflammatory markers, since WBC and ANC continue to increase from admission to 72 h post‐ICH [17, 19]; similarly, CRP showed a significant peak in the period over the 48 to 72 h [16]. The studies also suggest that the later values of CRP and not the admission ones, better correlate with the hematoma volume, with the functional outcome [16, 18]. Agnihotri et al [17] also point out to the value of the later (48–72 h) measurements of inflammatory markers post‐ICH as being more precise for neuroinflammation assessment, since they can reduce the impact of the premorbid conditions.

In this study, we also followed the temporal changes in WBC, CRP, and ESR during the disease course. Our results were in good congruence with the previously published data; all three inflammatory markers peaked 3 days post‐ICH, being highly significantly (*p* < 0.001) elevated, when compared to the admission values. At admission, leukocytosis was detected in only 17.8% of the patients, whereas on the 3rd day (72 ± 2 h post‐ICH)—57.5% of the patients had leukocytosis. Similar to the results of previous studies [16, 18], the later values for WBC, CRP and ESR (3rd day) correlated stronger with the neuroimaging markers (initial hematoma volume, volume of the edema) and with the neurological state at admission (CSS score), suggesting the benefit of their evaluation 72 h post‐ICH.

Concerning the different types of WBC, we detected neutrophilia in 62.5% of the patients 3 days post‐ICH. ANC correlated strongly with the neurological state on admission (CSS scores), with the initial volume of the hematoma and with the edema volume, which can be supported by previous studies [19, 20].

As regards to lymphocytes, lower number of ALC and lymphopenia were detected in 43.7% of our patients. Hitherto, several studies also reported higher neutrophils associated with lower lymphocytes in the post‐ICH setting [20]. Consequently, the neutrophil‐to‐lymphocyte ratio (NLR) was suggested as easy obtainable biomarker for systemic inflammation after ICH by some studies [20, 21]. High NLR values were confirmed to be associated with early hematoma expansion [21], neurological deterioration and poor functional outcome [20]. In our study, NLR strongly correlated with the neuroimaging markers (hematoma volume, and especially edema volume).

Last, in this study we also reported high inter‐dependence among the non‐specific inflammatory markers, detected by strong correlations among CRP, WBC, ESR, ANC, AMC and NLR, thus suggesting their interplay and possible synergistic effects in the promotion of the post‐ICH inflammation.

### 4.2 Pro‐inflammatory and anti‐inflammatory cytokines (IL‐6 and IL‐10): Inter‐dependence and correlations with clinical and neuroimaging markers

IL‐6 as important pro‐inflammatory mediator, was in the focus of brain injury research in the past decade; IL‐10 as anti‐inflammatory cytokine was shown to affect TGFβ production, thus having a protective role in ICH [22]. Nevertheless, when compared to the ischemic stroke, only several studies analyzed IL‐6 and IL‐10 in the ICH setting, and only a few showed correlations with neuroimaging variables.

As with the non‐specific inflammatory markers, clinical studies suggest later evaluation of ILs in ICH, as the peak of IL‐6 was detected over the 1st day to the 7th day [23], while the increase of IL‐10 started 2 days after ICH [24, 25]. In compliance with these findings, in our study IL‐6 and IL‐10 were evaluated 72 ± 2 h post‐ICH.

Dziedzic et al. [25], showed that ICH triggers release of both IL‐6 and IL‐10 in the peripheral blood, 2 days post‐ICH; the study also showed that serum IL‐6 correlates with the initial neurological state, with the volume of the hematoma and with the mass effect. A highly significant correlation was detected between IL‐6 and IL‐10 [25].

Our study demonstrated for the first‐time strong correlations of IL‐6 (3rd day) with the rest inflammatory markers on the 3rd day (WBC, CRP, ESR, ANC, AMC, NLR, and LMR) after ICH, which points out to their inter‐relation and inter‐dependence in the promotion of the inflammatory cascade. IL‐10 showed negative correlations with the rest inflammatory markers on the 3rd day (WBC, CRP, ESR, ANC, NLR, and IL‐6), suggesting attenuating effect on IL‐6 levels, but also possible effect on leukocytosis reduction (by decreasing WBC, ANC, and NLR).

### 4.3 OS markers and their relation to inflammatory markers

OS, that is, the accumulation of ROS after ICH plays important role in the secondary brain injury. Many studies argued intercalation between OS and neuroinflammation, since ROS can alter the post‐ICH inflammatory response via ROS‐induced activation of the NLRP3 inflammasome resulting with IL‐1β release, which can additionally promote neutrophil infiltration and perihematomal ROS production [26]. Microglial cells (activated by Hb) are also involved in this “vicious cycle,” which leads to massive ROS production and pro‐inflammatory cytokines synthesis [26, 27].

Experimental animal studies of ICH showed clear effects of OS on secondary brain injury post‐ICH via three mechanisms: progressive lipid peroxidation, (detected by higher MDA levels) [28], progressive protein oxidation (detected by carbonyl formations) [29], and inactivation or destruction of the enzymatic systems for ROS scavenging as a result of strong OS (detected lower activity of Mn‐SOD and CuZn‐SOD) [30]. Up to know, several clinical studies confirmed these mechanisms in ICH patients [31, 32]; however, hitherto an association of the OS markers with the clinical variables, or with the neuroimaging data was not shown [32].

Our results for the first‐time showed positive connection between inflammation and OS in a clinical study for ICH, detected by the positive and strong correlations between CRP‐AOPP, CRP‐CAT, IL‐6‐AOPP, IL‐6‐CAT, and negative correlation between IL‐10‐AOPP. These results show association between increased catalase activity and oxidized proteins levels with the pathological conditions of increased CRP, IL‐6, WBC, and low IL‐10 in ICH. For the first time we demonstrated that elevated OS (detected by high CAT activity and high AOPP) correlated with worse neurological state at admission (lower CSS scores). Last, protein oxidation (AOPP) showed correlation with neuroimaging variables (initial hematoma volume, and edema volume).

### 4.4 Main drivers in the formation of the brain edema

The overall analyses in this study revealed complex pathology in the edema formation post‐ICH; 18 variables correlated significantly with the edema volume and both significant synergistic and antagonistic effects of interaction among them were revealed. The final multiple regression model separated the 3rd day variables NLR, the moderator CRP × AOPP, and WBC as significant prognostic risk factors for formation of the edema volume along with the admission heart rate; IL‐6 was also included in the overall predictive capacity in the model, although not estimated as significant predictor.

The comprehensive analyses of our study, supported by previous animal and clinical studies, point out to several mechanisms in the formation of the edema: (i) leukocytosis and infiltration of WBC in the perihematomal tissue [26]; (ii) infiltrating neutrophils, which have direct effects on disruption of the blood‐brain barrier (BBB), microglial activation and neuronal apoptosis [33]; (iii) CRP‐induced disruption of the ВВВ, via binding to its Fcγ receptors on brain endothelial cells of the ВВВ [34]; (iv) interleukin synthesis [35]; (iv) OS, which can operate via several mechanisms, as lipid peroxidation [28], erythrocyte‐induced brain injury and protein oxidation [30], and Fe‐induced OS [29].

The results from the moderation analyses disclosed that these mechanisms do not operate alone with a simple additive effect, but synergistically in the propagation of the inflammatory cascade. For instance, the IL‐6 effects on the edema volume were moderated by NLR and ANC, suggesting that IL‐6 alone—without intensive neutrophil infiltration ‐ has a poor effect on the brain edema. Moreover, the neutrophil effects (ANC) on brain edema were enhanced in the conditions of high CRP.

Although we identified high positive AOPP correlation with the edema suggesting a role of ОЅ in edema formation, we also detected significant negative moderation effect of AOPP on the strength of the CRP‐edema dependence. The effect of CRP (3rd day) on the edema volume (5th day) was high only under low AOPP levels; high AOPP levels resulted with disruption of the edema dependence from CRP. The interaction plot (Figure 3E) summarizes that under high AOPP levels (484.6 μmol/L) and high CRP levels (over 300 mg/L), CRP does not contribute to brain edema formation at all, which indicates that in this pathological state, CRP is present, but its physiological effects are blunted, suggesting its inactivation. This “buffering” effect of protein oxidation on the physiological effect of CRP can be supported by several studies.

The function of CRP is controlled by a disulfide bond which acts as conserved redox switch; the expression of the pro‐inflammatory effects of CRP on brain endothelial cells is influenced by redox‐sensitive conformational changes, i.e., reduction of the disulfide switch [36]; contrarily, the oxidized CRR is inert in the activation of the endothelial cells and cannot bind to their Fcγ receptors. Jiang et al. [37], also reveal that in conditions of high CRP levels, the oxidation of the redox switch results in formation of cross‐links, binding to plasma albumin and leading to protein aggregation, which clearly attenuates its pro‐inflammatory effects.

These findings can explain the observed AOPP‐moderation effect on the dependence of the edema from CRP, taking into account that during high protein oxidation (detected by high AOPP), CRP is inactivated by oxidation, suggesting a potential OS‐CRP negative feedback loop in the mechanisms of brain edema formation.

Last, we would like to stress that our study procedures included multilevel comprehensive analyses of clinical and neuroimaging data, aligned with the exploration of 19 inflammatory and five OS markers; nevertheless, it should be considered that except these markers within the frame of our study, other markers have been also reported to have a role in post‐ICH brain edema formation. For instance, recent studies also show that matrix metalloproteinases are up‐regulated after ICH and play a crucial role in secondary brain damage and edema formation [38]. Although matrix metalloproteinases were not analyzed in this study, the opportunity to include such biomarkers in prediction models of edema formation may open ideas for further research.

### 4.5 Study limitations

As most of the studies focusing on ICH, our study has limitations. The data was derived only from two clinics (University Clinic of Neurology and, University Clinic of Neurosurgery, Skopje), including patients with Macedonian ethnicity. The results of this study are also limited only to conservatively treated patients without other complications during the disease course, as intraventricular and subarachnoid bleeding, or hematoma expansion. Last, catalase activity and advanced oxidation protein products are not routinely examined in the clinical practice and these parameters are not available in most of the stroke centers, which might limit the formula for quantification of the edema for a widespread application. Nevertheless, possible strategies to overcome this limitation is to use our second developed model without the moderation CRP × AOPP effect, resulting in a formula (given below) that has lower predictive capacity (*R* ^2^_adj_ of 58.6%), but it can be more widely applied in the clinical practice.

Adjusted formula (second multiple regression model without the CRP × AOPP moderation effect; lower R^2^_adj_ of 58.6%):

*V*(edema, 5th day) = 2.666∙(NLR 3rd day) + 0.429∙(admission heart rate) + 1.881∙(WBC 3rd day) + 0.180∙(IL‐6 3rd day) – 2.486 (admission CSS) + 0.057 (CRP 3rd day) + 2.397.

## 5 CONCLUSIONS

We consider that the main contribution of our study is the generated mathematical model with 3D graphical plots for prediction of the volume of the edema, which allow its quantitative prognostication on the basis of admission and 3rd day variables; the mathematical model also takes into consideration the interplay and the detected synergistic and antagonistic interactions among the significant and non‐significant predictors in the model. In conclusion, we suggest consideration of our mathematical equation as a tool for prediction of the edema volume, which might be beneficial for taking time‐adequate medical strategies for attenuation of the brain edema detrimental effects, thus helping in prevention of the delayed neurological deterioration in these threatened patients.

## AUTHOR CONTRIBUTION

Vladimir Rendevski designed the study, performed all of the initial screening for eligibility and defined the cohort group of patients for the study; he also performed the neurological (clinical) examinations with Nensi Manusheva and they wrote the sections Introduction and Methods. Boris Aleksovski did the complete biochemical part of the study; he performed the basic biochemical measurements and the measurments of the inflammatory markers. Boris Aleksovski have also performed the complete statistical analyses of the study and he wrote the sections: Results, Discussion and Conclusions. Ana Mihajlovska Rendevska and Blagoj Shuntov performed the CT scans and summarized all the neuroimaging data. Nikola Hadzi‐Petrushev and Icko Gjorgoski performed the biochemical assays for estimation of the oxidative stress markers and gave intellectual input for their interpretation.

## FUNDING INFORMATION

No funds, grants, or other support was received for conducting this study.

## CONFLICT OF INTEREST

The authors report no conflict of interest.

## ETHICS STATEMENT

This study has been performed in accordance with the ethical standards laid down in the 1964 Declaration of Helsinki and its later amendments. The study was approved by the Human Research Ethics Committee within UKIM‐Medical Faculty in Skopje.

## CONSENT TO PARTICIPATE

A written informed consent was obtained from all subjects before the inclusion in the study.

## CONSENT FOR PUBLICATION

All authors have approved the version to be published.

## Supporting information


**FIGURE S1** Validity and overall fit of the multiple regression model for prediction of the edema volume.

## Data Availability

The original research data are available upon request.
