# Supplementary material for: Inflammatory and oxidative stress markers in intracerebral hemorrhage: Relevance as prognostic markers for quantification of the edema volume
Source: Brain Pathol. 2022 Jun 28;33(2):e13106. doi: 10.1111/bpa.13106 (PMC10041164; doi:10.1111/bpa.13106)
Supplement: Supplementary file 1 — FIGURE S1 Validity and overall fit of the multiple regression model for prediction of the edema volume. [file BPA-33-e13106-s001.pdf]

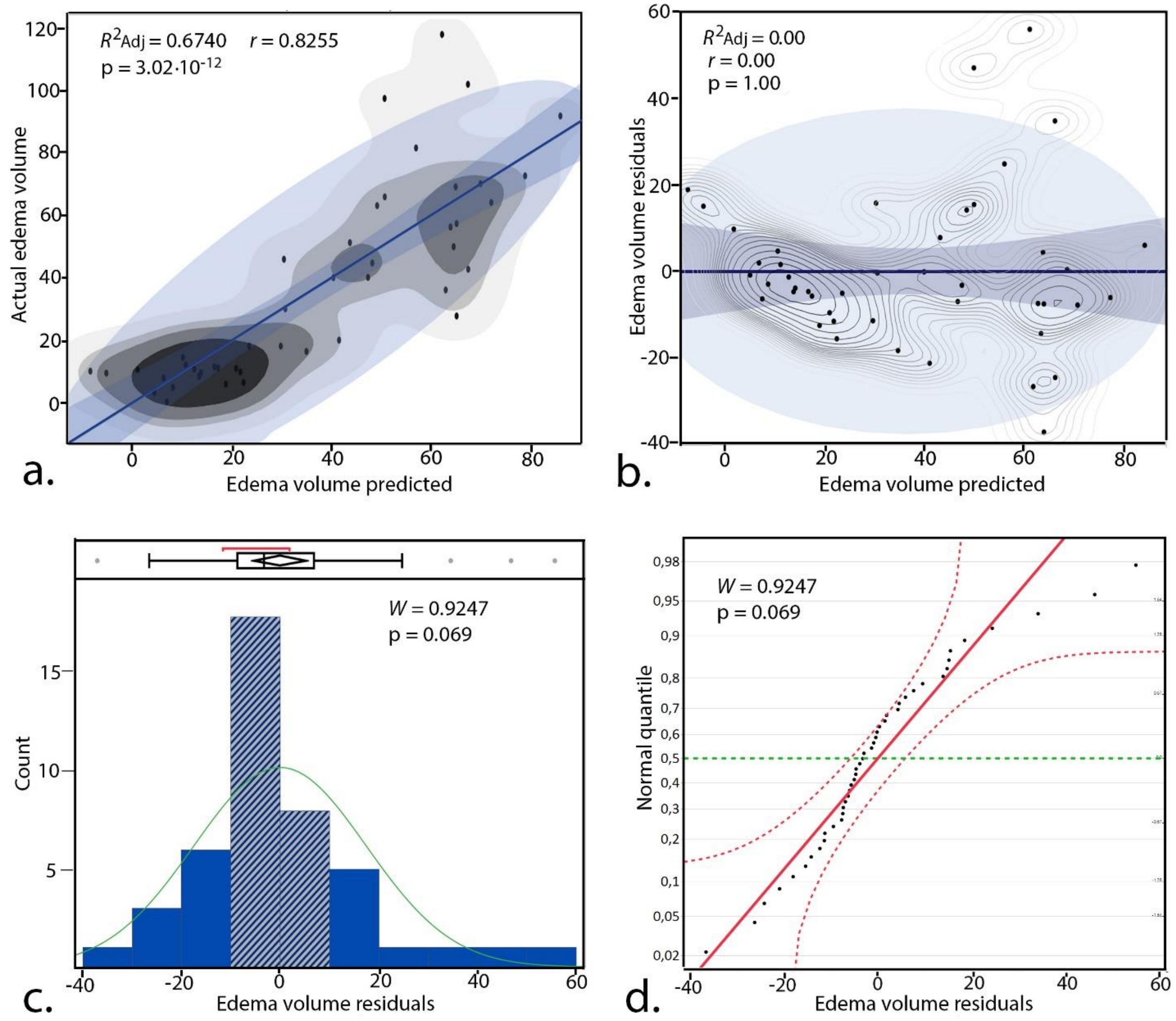

Supp. Fig. 1. Validity and overall fit of the multiple regression model for prediction of the edema volume.

a. High and significant correlation between the actual and predicted edema volume (r = 0.8255; R^2_{adj} = 0.6740, F = 76.2860, ***p = $3.02 \cdot 10^{-12}$), suggesting good overall fit of the model.

b. The homoscedasticity assumption was checked by plotting the standardized residuals versus the predicted edema volume and the scatterplot showed equal distribution across all values of the independent variable (evident absence of any correlation between the residuals and the predicted edema volume: r = 0.0; R^2 = 0.00, F = 0.00, p = 1.00).

c-d. Approximately normal distribution of the residuals, additionally proven by the Shapiro-Wilk test (W = 0.9247, p = 0.069).
